# Rice fields along the East Asian-Australasian flyway are important habitats for an inland wader’s migration

**DOI:** 10.1038/s41598-020-60141-z

**Published:** 2020-03-05

**Authors:** Satoe Kasahara, Gen Morimoto, Wataru Kitamura, Sadao Imanishi, Nobuyuki Azuma

**Affiliations:** 1 Suwa Hydrobiological Station, Faculty of Science, Shinshu University, 5-2-4 Kogan-dori, Suwa Nagano, 392-0027 Japan; 2 Institute of Mountain Science, Shinshu University, 5-2-4 Kogan-dori, Suwa Nagano, 392-0027 Japan; 3 Faculty of Agriculture and Life Science, Hirosaki University, Bunkyo-cho 3, Hirosaki Aomori, 036-8561 Japan; 4 Division of Avian Conservation, Yamashina Institute for Ornithology, Konoyama 115, Abiko Chiba, 270-1145 Japan; 5 Faculty of Environmental Studies, Tokyo City University, Ushikubo-nishi 3-3-1, Tsuzuki-ku, Yokohama, Kanagawa 224-8551 Japan; 6 Tatemachi 493-1, Hachioji Tokyo, 193-0944 Japan

**Keywords:** Animal migration, Wetlands ecology

## Abstract

To maintain and recover populations of migratory waders, we must identify the important stopover sites and habitat use along migration routes. However, we have little such information for waders that depend on inland freshwater areas compared with those that depend on coastal areas. Recent technological developments in tracking devices now allow us to define habitat use at a fine scale. In this study, we used GPS loggers to track both spring and autumn migration along the East Asian-Australasian flyway of the little ringed plover (*Charadrius dubius*) as birds moved to and from their breeding grounds, gravel riverbeds in Japan. The birds we tracked overwintered in the Philippines and made stopovers mainly in Taiwan and the Philippines. The most important habitat during the non-breeding season was rice paddy fields. Our findings imply that changes in agriculture management policy in the countries along the migration route could critically affect the migration of waders that depend on rice paddy fields. To maintain populations of migrant inland waders that move within the East Asian-Australasian flyway, it is necessary not only to sustain the breeding habitat but also wetlands including the rice paddy fields as foraging habitat for the non-breeding season.

## Introduction

Migrant waders have been decreasing on a worldwide scale^1–5^ due to various complex reasons^6^, such as land-use change^2^ and increasing human activity^4,5^. Since these migrant birds use multiple habitats and climatic zones within their annual life cycle^7^, identifying the migration pattern and route, including the habitats used along the way, is imperative for understanding the factors that affect population changes and/or contribute to efficient conservation activities. The loss or degradation of stopover sites is a particularly serious issue^1,2,4–6^. In recent decades, therefore, many studies have investigated the migration patterns, detailed migration routes, and important stopover sites for several waders by using tracking methods ranging from traditional color flagging to advanced systems such as satellite and light-level geolocators^8–12^.

Studies of migration ecology of waders, however, have mainly focused on species that depend on coastal areas (i.e., shorebirds), and there is still little information on those that depend on inland freshwater areas. The low visibility of inland waders compared with that of coastal waders and the coarse spatial resolution of previously used tracking devices (light-level geolocators^13^, 50–200 km; Argos system, <150 m under good conditions and >1000 m under worse conditions^14^) made it difficult to determine the fine-scale habitat use of individuals. However, inland natural wetlands have been dramatically declining on a global scale^15^, and some studies in the East Asian-Australasian flyway (EAAF) reported that waders that depend on inland habitat such as rice paddy fields have been decreasing^2,16^. For migrant birds, rice paddy fields function much like semi-natural wetlands^17^, and rice is grown across a wide range of landscape types in East and Southeast Asia^18^. Therefore, it is important to survey the fine-scale habitat use in inland waders to assess the relative importance of artificial habitats such as rice paddies for sustaining these bird populations during migration. In this study, we used GPS loggers to track several migrating little ringed plovers (*Charadrius dubius*) during their spring and autumn migrations. This advanced tracking device can record the longitude and latitude directly with high accuracy (<10 m)^19^, particularly in an open landscape^20,21^ and/or with more satellites^21^. Thus, this system allowed us to delineate the whole migration route and to track fine-scale habitat use during the migration period.

The little ringed plover is a small migrant wader that mainly inhabits inland open freshwater areas^22^ and is threatened primarily by the degradation and loss of its preferred habitats^23^, although the species is classified as least concern in the IUCN red list^24^. Among the three subspecies of the plover (*C. d. curonicus, C. d. dubius*, and *C. d. jerdoni*)^23^, *C. d. curonicus* breeds across a wide range of the Palearctic including Europe and Japan; southern overwintering areas include sub-Saharan Africa, parts of the Arabian Peninsula, eastern China, and Indonesia^23,25^, as well as Southeast Asia^26,27^. The migration ecology and route of the European *C. d. curonicus* population has been clarified by bird banding^22^ and by tracking using geolocators^28^. However, little is known about these aspects of the Asian population that move within the EAAF, although some information suggests the potential importance of several regions in the non-breeding season (Thailand, the Philippines, Taiwan, and Myanmar) and during migration (Thailand, Russia, and eastern China)^26^. Regarding the migration route of the Japanese population, the only record is that of a plover released from Taiwan that was recaptured in central Japan^29^. The little ringed plover is a relatively common species in Japan, however, breeding habitat—bare gravel-covered ground near water such as rivers^22^—is rapidly disappearing or degrading due to expansion of vegetation and trees, which has resulted in less natural flooding^30,31^. Thus, the aim of this study was to identify the important stopover and wintering sites and the fine-scale habitat use of the little ringed plover, an inland wader, to help reduce the risk of future population decline.

## Results

We attached GPS loggers to 19 (10 males, 9 females) breeding plovers in 2017 and recaptured 6 of them (31.6%, 3 males, 3 females) in 2018 at the same breeding site in central Japan. On recapture, we found bald spots on the skin where the GPS logger was attached and on the thigh around the harness but noted no scar or abrasion on the skin. Although the body mass of recaptured birds had decreased (mean decrease ± SD: 1.8 ± 2.1 g, *n* = 6), the total body weight did not change significantly over the course of migration to and from the breeding site (*t* = 2.10, df = 5, *P* = 0.09, matched pairs *t*-test).

Of the six recaptured birds, two had complete data including wintering site and autumn and spring migration routes. Two others had incomplete data for spring migration because the GPS logger stopped working. The remaining two birds had only autumn migration data until mid or late October because the GPS logger stopped or the antenna for position fixing was lost.

**Figure 1:**
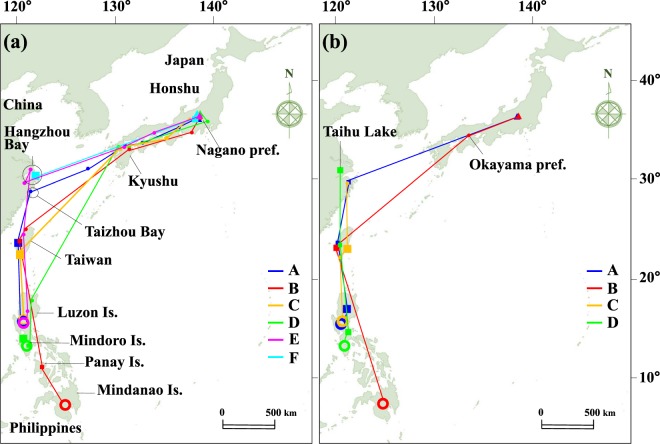
Autumn (**a**) and spring (**b**) migration tracks of six little ringed plovers recorded by GPS logger. Triangles and large open circles indicate breeding sites and wintering areas, respectively. Small circles show transit points where birds stayed for fewer than 4 days (i.e., the GPS fixing interval), and squares indicate stopover sites where they stayed for more than 4 days. The loggers of plovers E and F stopped on October 17 and October 29 in 2017 and those of plovers C and D stopped on March 18 and March 25 in 2018. The map was made from Natural Earth map (http://www.naturalearthdata.com).

The six plovers started the autumn migration in late June to mid July in 2017, after natural flooding by heavy rain washed over the breeding ground and put a stop to their breeding. Plovers A–E arrived at their respective wintering areas from early August to mid November (Table 1), and plover F followed a different path, discussed in the next paragraph. After leaving the breeding sites, plovers A–E passaged within Honshu in Japan for 8–16 days without a stopover of more than a GPS fixing interval (4 days, see Methods) and migrated abroad via Kyushu, located in the southern part of Japan (Fig. 1a, Table 1). Then, plovers A and E visited coastal areas near Hangzhou Bay or Taizhou Bay in eastern China and then separately flew to western Taiwan. Finally, these two plovers moved to the wintering area in central Luzon Island in the Philippines at different times. Although the GPS logger of plover E stopped in late October, its location was likely to be the wintering area of the bird because it had stayed near there for almost 3 months and the other three plovers had already arrived at their wintering areas by then. In addition, plovers A and C overwintered in a nearby region. Plovers B and C moved to wintering areas in central Luzon Island or northern Mindanao Island in the Philippines via western and southern Taiwan after leaving Kyusyu. Plover B also visited Panay Island before moving to its wintering area. Plover D reached its wintering area, north Mindoro Island, via Luzon Island after leaving Kyusyu. During the autumn migration, Plovers A, B, and C made a stopover in Taiwan for 4–80 days (Table 1). Plover D reached its wintering area in mid July, however, the bird moved northward to southern Luzon Island and stayed there from mid August to mid November before flying back to northern Mindoro Island. The autumn migration distances of the five birds that went to the Philippines were 3108–4226 km and the durations were 32–136 days. Detours from the direct route accounted for 8.1–25.7% of the total distance. The minimum and maximum migration distances per 4 days (i.e., the GPS fixing interval) were 51 and 1944 km, respectively.

**Table 1:** Migration data of six little ringed plovers (*Charadrius dubius curonicus*) tracked by GPS loggers.

	Plover A Female	Plover B Male	Plover C Male	Plover D Female	Plover E Male	Plover F Female
Tracking period (days)	320	304	292	284	136	112
**Autumn migration in 2017**						
Start of migration	20 Jun.	2 Jul.	12 Jul.	29 Jun.	1 Jul.	15 Jul.
Stopover days in Japan	<4	<4	<4	<4	<4	16
Stopover days in China	<4	—	—	—	<4	74*
Stopover days in Taiwan	80	4	56	—	<4	—
Stopover days in the Philippines	<4	4	<4	120	<4	—
Total duration (days)	108	32	76	136	32	(20)
Migration distance (km)	3267	4226	3108	3673	3601	(1658)
Direct distance (km)	2854	3520	2875	3103	2865	(1650)
Detour (%)	14.5	20.1	8.1	18.4	25.7	(<1)
**Wintering period**						
Wintering area	Luzon Is.	Mindanao Is.	Luzon Is.	Mindoro Is.	Luzon Is.	(Daishan)
Arrival	6 Oct.	3 Aug.	26 Sept.	12 Nov.	2 Aug.	(4 Aug.)
Duration (days)	104	196	144	80	88*	—
Travel distance (km)	194	162	218	64	116*	—
**Spring migration in 2018**						
Start of migration	18 Jan.	15 Feb.	17 Feb.	31 Jan.	—	—
Stopover days in the Philippines	44	<4	<4	16	—	—
Stopover days in Taiwan	12	16	20	4	—	—
Stopover days in China	4	—	<4*	12*	—	—
Stopover days in Japan	<4	<4	—	—	—	—
Total duration (days)	72	32	36*	44*	—	—
Arrival at breeding site	31 Mar.	19 Mar.	—	—	—	—
Migration distance (km)	3303	4125	1581*	1993*	—	—
Direct distance (km)	2863	3519	1551*	1971*	—	—
Detour (%)	15.4	17.2	1.9*	1.1*	—	—

The loggers of plovers E and F stopped on October 17 and October 29 in 2017 and those of plovers C and D stopped on March 18 and March 25 in 2018, respectively. Dashes indicate a lack of data because the bird did not pass through a region or the logger had stopped. Asterisks indicate a limited record until the GPS logger stopped. Because it is uncertain whether the area where plover F finally stayed was the wintering area, some information of the bird is given in parentheses.

Only plover F made a stopover for 16 days in Honshu, at a site 30 km southwest of the breeding site. Then, the plover moved to Daishan in Hangzhou Bay of eastern China without any other stopover in Japan. Although the plover stayed there until mid October, subsequent movement was unclear because the GPS logger’s antenna was lost. We lacked the evidence to determine whether the area where plover F stayed was its wintering area or not, except that it coincided with the arrival at wintering areas of the other plovers. The moving distance and duration of plover F, which migrated to China, were 1658 km and 20 days.

The wintering period of four plovers (A–D) was 80–196 days, and the minimum and maximum trip distances per 4 days during overwintering were less than 1 and 49 km, respectively. The four plovers started the spring migration between mid January and mid February. Plovers A and B returned to the breeding site in mid and late March (Table 1). After leaving the wintering area, plover B moved to southwestern Taiwan and made a stopover there for 16 days, and then returned to the breeding site via Okayama prefecture, Japan (Fig. 1b, Table 1). Plovers A and D made a stopover on Luzon Island for 44 and 16 days, respectively, before moving to Taiwan and they also separately made stopovers in southwestern Taiwan. The two plovers then moved inland near Hangzhou Bay or Taihu Lake in eastern China and made a stopover there; however, the GPS logger of plover D then stopped working. Plover A departed for Japan after a 4-day stopover in China and returned to the breeding site without any stopover lasting more than 4 days. Plover C moved to Taiwan after leaving the wintering area without any stopover, and it remained in southeastern Taiwan for 20 days. The plover then moved inland near Hangzhou Bay in eastern China, but the GPS logger stopped there. The spring migration distances of the two plovers with complete data were 3303 and 4125 km, and the durations were 72 and 32 days. Detours from the direct route accounted for 15.4% and 17.2% of the total distance, and the minimum and maximum migration distances per 4 days (i.e., the GPS fixing interval) were 89 and 1837 km.

**Figure 2:**
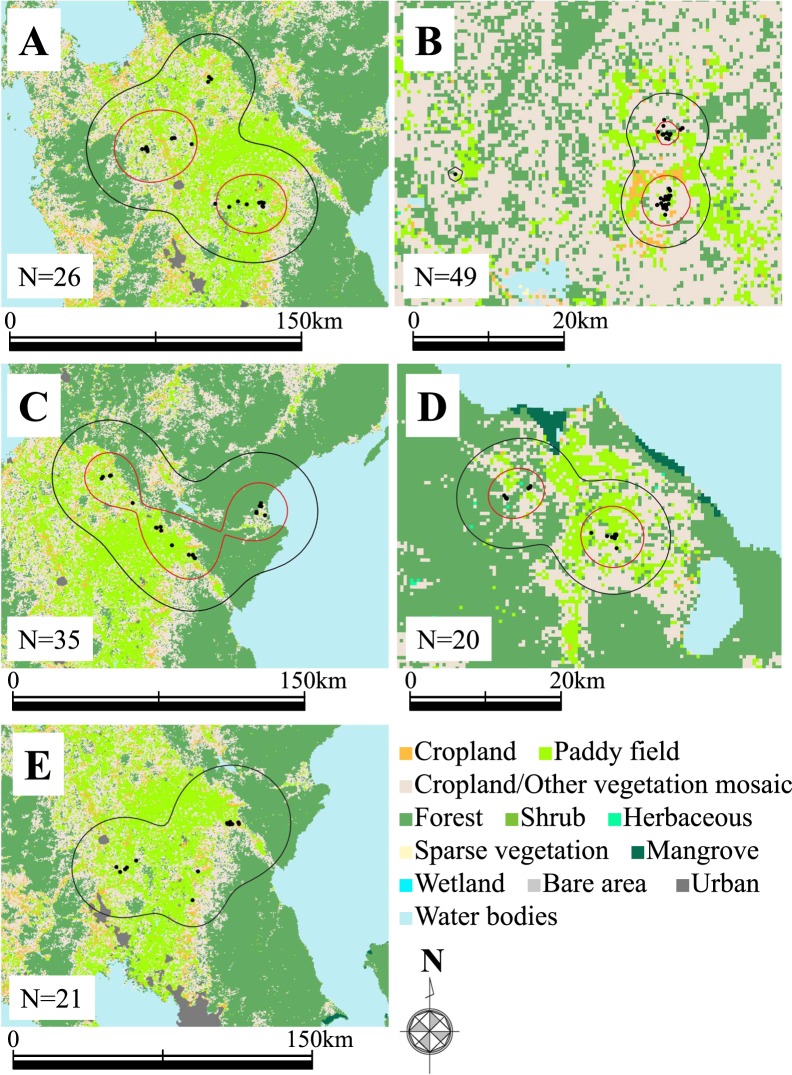
Maps of wintering home ranges for five little ringed plovers. (**A–E**) Indicate plover IDs. Black points and N values indicate certain fixing position by GPS logger and the number of them during the wintering period, respectively. Black and red lines mark the 95% and 50% kernel density estimates of wintering home range, respectively. Because plover E had limited data until the GPS logger stopped (see Table 1), only the 95% kernel density estimate is shown. The map was modified from the Global Land Cover data version 3^56^ developed by national mapping organizations (GLCNMO) which is constituted from Geospatial Information Authority of Japan, Chiba University and collaborating organizations. We modified some land cover categories from original version (see Methods).

**Figure 3:**
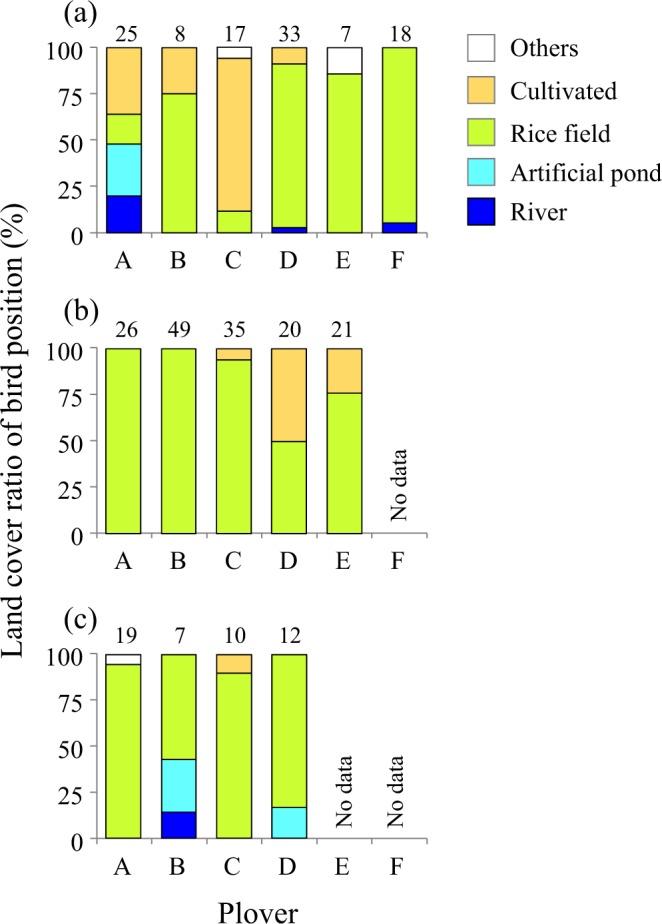
Land cover ratio of bird positions fixed by GPS logger during autumn migration (**a**), wintering season (**b**), and spring migration (**c**). The number of certain fixing positions by GPS logger at an altitude less than 100 m is shown above each bar. Artificial pond included culture farm and cultivated area included croplands and orchards.

In the wintering period, the home ranges of little ringed plovers were established in inland areas. Mean wintering home range size and core range size (Fig. 2A–E) by the 95% and 50% kernel density estimates were 4447 ± 3878 km^2^ (mean ± SD, range: 209.9–7918 km^2^, *n* = 5) and 1121 ± 1223 km^2^ (mean ± SD, range: 43.6–2357.6 km^2^, *n* = 4), respectively. Plovers A, B, and D had two separate core areas, even though two plovers (B and D) had small home ranges. Most of the locations recorded by GPS for the five plovers were rice paddy fields. The result of environmental analysis using more detailed land cover information (see Methods) indicated that paddy rice field was the most common land cover type where plovers were recorded throughout the non-breeding season. In the autumn migration period, the mean recorded position ratio of rice paddy fields was 61.8 ± 37.6% (mean ± SD, range: 11.8–94.4%, *n* = 6, Fig. 3a), followed by areas cultivated in other crops (25.4 ± 31.4%, 0–82.4%, *n* = 6). The ratio of paddy rice field became higher in the wintering period (Fig. 3b, 84.1 ± 21.4%, 50–100%, *n* = 5) and spring migration period (Fig. 3c, 81.3 ± 16.8%, 57.1–94.7%, *n* = 4). During migration, some plovers often used wetlands, including artificial ponds of bivalve farm and rivers, mainly in Taiwan but also in China and Japan.

## Discussion

Our tracking study provided three main findings about the migration of the Asian population of an inland wader, the little ringed plover. First, the tracked plovers that bred in Japan overwintered across a wide range of the Philippines. This is direct evidence that the country is a wintering ground of the subspecies *C. d. curonicus*. Previously, Southeast Asia had been included in the distribution ranges of *C. d. curonicus*^25,27^ and the Philippines is known as a distribution area of *C. d. jerdoni*. The Ornithological Society of Japan’s domestic bird lists, since the 6th revised edition^32^, has included the Philippines as a wintering area of the subspecies we tracked, although the source record of that description was unclear. Thus, our study provides the first strong evidence that Japanese populations of *C. d. curonicus* overwinter in the Philippines.

Second, we identified some stopover sites of this species. Taiwan was a common stopover site for the plovers we studied during both the autumn and spring migrations (Table 1, Fig. 1), which corresponds with a previous ringing record in Japan^29^. Some plovers also made a stopover in the Philippines just before and after the wintering season. The post-breeding molting stage sometimes begins in June in this species^22^, which could explain these stays for more than 1 month, as well as the birds’ need to obtain fuel for migration. Thus, both countries are important for the migration of the little ringed plover as well as other waders in both coastal and inland habitats in the EAAF^9,33^. The plovers we tracked adopted short flights with multiple transits during the autumn and spring migrations. Our data also suggest that the birds need to obtain resources for migration and other annual cycle stages (e.g., molt) in these countries. A similar migration pattern of the little ringed plover has been reported in the local population of Sweden^28^ and in other small shorebirds in the EAAF, such as sanderling^34^ and ruddy turnstone^8^. However, the migration routes and wintering areas of the six plovers we studied had less variability compared with the Swedish population, which showed a variety of migration routes among seven studied plovers. In addition, the migration routes and distances of the two plovers with complete migration data were similar in autumn and spring, as has been reported in sanderling^34^. The birds’ small body size may restrict their selection of favorable stopover sites, with the sea acting as an ecological barrier. Although plovers made stopovers at only a few sites in Japan and China, the relatively long position-fixing interval of 4 days that we set for the GPS logger may have obscured some stopover sites in eastern China and Kyusyu, Japan, where the plovers could refuel before a long-distance flight. More intensive tracking is needed in the future.

Our third finding was that paddy fields are the most important habitat for the little ringed plover during the migration and wintering periods (Figs. 2 and 3). Although rice paddy fields account for only 21% and 32% of the total farmland area in Taiwan and the Philippines^18,35^, respectively, rice paddies represented 62–84% of the land cover visited by the plovers. These data suggest that plovers select rice paddy fields as stopover habitat over other land cover types. In addition, although the kernel density estimates showed that the area of wintering home range varied among individuals, some of them had multiple small core areas. These findings suggest that little ringed plovers tend to stay in particular areas or narrow ranges. The double cropping method used in these countries^36,37^ could foster such circumstances by providing suitable foraging habitat—areas with low vegetation and shallow water—to the plovers throughout the year. Other studies have also reported the importance of paddy fields for migrating or wintering bird species^2,16,38^. Amano *et al*.^2^, however, suggested two possible factors that have effected on decline of wader population dependence on rice paddy fields: (1) reduced availability of prey such as aquatic insects due to the introduction of efficient drainage systems to make rice paddy fields dry and (2) decreased rice paddy field area and/or areas with short vegetation, which is favorable foraging ground for waders, due to diversion of agriculture from rice to dryland crops and increased tall and dense vegetation area in fallow fields. Thus, changes in the management of paddy fields in the countries along the migration route could critically affect migration of the plover species that make stopovers there. In fact, the area of rice paddy fields has already declined in Taiwan due to an increase in fallow fields^39^ and diversion to forage crops^40^. Moreover, climate change could be a new threat. Although the area of rice paddy fields has been increasing for decades in the Philippines, future air temperature rise could restrict rice production in the country^41^. Reduction of the species’ habitat range due to a shift in land use or climate change could affect the birds’ fitness in subsequent locations and the long-term population dynamics^42^. To detect and reduce such effects, it is necessary to gather data on the migration route and habitat use of many more inland waders.

Habitat use of the little ringed plover clearly differed between the breeding and migration/wintering periods, as is common in migratory birds^43^. Our findings indicate the need to maintain both major habitats, gravel-covered ground and rice paddy fields, to sustain the plover species, in contrast with a previous study that indicated the species uses rice paddy fields less frequently in Japan^44^. Of course, other wetlands such as culture farm pond and rivers also should not be ignored because habitat usage and flight pathways varied among individuals.

In the case of the two plovers for which we had both autumn and spring migration data, the birds returned to the breeding site while stopping at many fewer transit points in Japan than during the autumn migration. They may move toward the breeding site over a shorter period due to site fidelity and to enhance their reproductive success^45^, which is influenced by particular breeding habitat characteristics and/or quality^46,47^. Therefore, maintaining suitable bare gravel ground in Japan should contribute to sustaining a breeding population of the little ringed plover.

In conclusion, our plover tracking data suggest that rice paddy fields are significant potential habitat for inland waders in the EAAF, in contrast with many previous studies that emphasized the importance of mudflats around estuaries such as the Yellow Sea, which is a primary stopover site for huge populations of coastal waders^2,4,48^. Thus, we need to consider the land use of both coastal and inland wetlands to conserve the populations of migrant waders in the EAAF. To conserve these species, it is important not only to maintain or restore breeding habitat, but also to maintain the habitats used by waders in the non-breeding season, which will require extensive collaboration among the various countries concerned.

## Materials and Methods

We captured breeding adults of the little ringed plover at the three sites along the middle course of the Chikuma River (36°29′58′′N, 138°07′51′′E; 36°40′47′′N, 138°16′40′′E; and 36°42′32′′N, 138°17′58′′E) in central Japan. Their egg-laying season is from late April to late June, and we trapped most of them in June 2017. Birds were captured by using a funnel trap^49^, fall trap, and bow net^50^ placed on the nests that were on the gravel-covered ground; the birds were recaptured in 2018 using the same traps. We measured body weight of captured birds before attachment of GPS logger (Pinpoint-10, 21 mm × 13 mm × 5 mm, Lotek Inc., Newmarket, Ontario, Canada) to ensure that the total weight of the device including the harness (1.3–1.4 g) was less than 4% of the bird’s body weight (mean ± SD: 3.4 ± 0.1%, range: 3.2–3.7%, *n* = 19). We attached a GPS logger to the back of birds using a leg-loop harness^51^ and programmed theses loggers to take a GPS fix every 4 days at 12:00 pm GMT from June 2017 onward. We also banded each captured bird using a unique combination of an aluminum ring and a color ring to allow for identification the following year even if the GPS logger was covered by feathers.

Lower foraging efficiency or increased energy expenditure were suggested as serious effects by tracking device attachment^52^. Thus, we measured body weight again after the GPS logger was removed from recaptured birds and checked whether their body had any injuries. To examine the foraging and energy costs due to the tracking device, we compared the body weight of birds before device attachment and after removed it, by matched paired *t*-test.

Although the GPS logger we used has mostly high accuracy in location fixing^19^, large errors sometimes arise depending on the number of satellites^21^, the strength of the satellite configuration, or geometry^20^. Thus, we checked the dilution of precision (DOP) value of the collected data, which indicates accuracy of GPS positioning; low DOP values (i.e., <1) indicate favorable accuracy and higher DOP values indicate increasingly poor satellite geometry at the time of the location fix^20^. Before analysis, we considered the data with DOP values of ≥ 4 as low accuracy^20^, resulting in the removal of 1–5 points from each plover’s tracking data, and the error ratio to the total number of fixing positions was 1.3–17.9% in each plover. We also removed another class of fixing positions from the analysis regardless of the DOP, namely where the bird was recorded in the sky at a height of more than 100 m above the ground. However, all fixing positions including points with higher DOP and higher sky location were involved in the calculation of the staying period in each country.

We calculated migration distance as the sum of distances between the recorded positions with lower DOP value. We defined the wintering area as the place where the plover made a stay of more than 2 months without long-distance flight (>50 km) toward a more southerly region between November and January. Likewise, we identified a stopover site as a region including at least two GPS-fixed positions (i.e., duration of stay at least 4 days) within 50 km. We defined the start positions of the autumn and spring migrations of each plover as the locations just before they moved more than 50 km from the breeding site where we attached the GPS tag and from the wintering area, respectively.

To understand which area and habitat character are more important for wintering plovers, we defined wintering home range and core area of five plovers that arrived at the wintering area by 95% and 50% kernel density estimates using QGIS (version 3.6.1)^53^ and the software package adehabitatHR (version 0.4.16)^54^ in R (version 3.5.1)^55^. The base map of the Fig. 2 was modified from the Global Land Cover data version 3^56^ developed by national mapping organizations (GLCNMO) which is constituted from Geospatial Information Authority of Japan, Chiba University and collaborating organizations. Although original version has 20 land cover categories^56^, we reclassified them, with the exception of snow/ ice category, into 12 categories to show environments of the points that plovers were recorded; original 6 forest types^56^ integrated into “Forest”, original 2 herbaceous types integrated into “Herbaceous”, two types of bare area into “Bare area”. In addition, to identify the detail habitat use of the six plovers during migration and wintering, we determined the land cover types of each position by using land cover data of the countries along the migration routes after 2000^57–62^, aerial photographs from Google Earth Pro, and a field survey in Taiwan in November 2018. We classified five land cover categories: (1) river, (2) artificial pond such as bivalve farms, (3) rice field, (4) cultivated (cropland or orchard), and (5) others, such as bare ground.

In the study, bird handling was carried out according to the banding manual of the Yamashina Institute for Ornithology^63^. The experiment protocol of bird trapping and GPS logger attachment was approved by the Ministry of the Environment, Japan, and we conducted them with legal permission from the ministry (1704191, 1804139) and the local government of Nagano prefecture (29-16-8, 30-7-2).

## Data Availability

All the position data of the six plovers gathered in the current study are available from the corresponding author on reasonable request.
